# Potential application and beneficial effects of a marine microalgal biomass produced in a high-rate algal pond (HRAP) in diets of European sea bass, *Dicentrarchus labrax*

**DOI:** 10.1007/s11356-021-14927-x

**Published:** 2021-06-29

**Authors:** Giulia Pascon, Maria Messina, Lisa Petit, Luisa Maria Pinheiro Valente, Beatriz Oliveira, Cyrille Przybyla, Gilbert Dutto, Francesca Tulli

**Affiliations:** 1 Department of Agricultural, Food, Environmental and Animal Sciences, University of Udine, 33100, Udine, Italy; 2 CIIMAR, Centro Interdisciplinar de Investigação Marinha e Ambiental, Universidade do Porto, 4450-208 Matosinhos, Portugal; 3 ICBAS, Instituto de Ciências Biomédicas de Abel Salazar, Universidade do Porto, Rua de Jorge Viterbo Ferreira, 228, 4050-313 Porto, Portugal; 4 MARBEC, Univ. Montpellier, CNRS, Ifremer, IRD, Palavas les flots, Laboratoire L-3AS, 34250 Palavas-les-Flots, France; 5 IFREMER French Research Institute for Exploitation of the Sea, Laboratoire Service d’Expérimentations Aquacoles, 34250 Palavas les flots, France

**Keywords:** Microalgae consortium, *Nannochloropsis* spp., *Oocystis* sp., Gut physiology, Diet digestibility, Water treatment, European sea bass

## Abstract

Microalgae have been used as live food in aquatic species. In recent years, the interest in microalgae has considerably increased, thanks to the evolution of production techniques that have identified them as an ecologically attractive aquafeed ingredient. The present study provides the first data about the effects of dietary inclusion of a microalgae consortium grown in a high-rate algal pond system on zootechnical performance, morphometric indices, and dietary nutrient digestibility as well as morphology and functionality of the digestive system of European sea bass, *Dicentrarchus labrax*. A dietary treatment including a commercial mono-cultured microalgae (*Nannochloropsis* sp.) biomass was used for comparison. Six hundred and thirty-six European sea bass juveniles (18 ± 0.28 g) were randomly allotted into 12 experimental groups and fed 4 different diets for 10 weeks: a control diet based on fish meal, fish oil, and plant protein sources; a diet including 10% of *Nannochloropsis* spp. biomass (100 g/kg diet); and two diets including two levels (10% and 20%) of the microalgal consortium (100 and 200 g/kg diet)*.* Even at the highest dietary inclusion level, the microalgal consortium (200 g/kg diet) did not affect feed palatability and fish growth performance. A significant decrease in the apparent digestibility of dry matter, protein, and energy was observed in diets including 10 and 20% of the microalgal consortium, but all fish exhibited a well-preserved intestinal histomorphology. Moreover, dietary inclusion with the microalgal consortium significantly increased the enzymatic activity of maltase, sucrase-isomaltase, and ɤ-glutamil transpeptidase in the distal intestine of the treated European sea bass. Algal consortium grown using fish farm effluents represents an attempt to enhance the utilization of natural biomasses in aquafeeds when used at 10 % as substitute of vegetable ingredients in diet for European sea bass.

## Introduction

Aquaculture plays a key role in supporting human nutrition (Olsen 2011), and the increased availability of raw materials for feed formulation is required to support its rapid and continuous growth. For this reason, research has been focused, for a long time, on finding alternative ingredients to the traditional ones used by the feed industry to reduce pressure on natural resources while addressing the growing market demand for aquaculture products. Management of sustainable feeding practices for aquatic organisms involves, both from a technical and an economic point of view, identifying alternative resources that consider the nutritional profile and the effects on animal welfare (resistance to stress and disease), while preserving the nutritional quality of the seafood product. It also implies the management of livestock activities waste into the environment (FAO 2018).

The use of microalgae as a potential ingredient of aquafeeds could represent an ecologically attractive alternative not only to traditional ingredients of marine and plant origin (Becker 2007) but also to innovative ingredients such as insects, seaweeds, and yeasts. In addition to their basic nutritional value (Spolaore et al. 2006), the inclusion of microalgae in aquafeeds is becoming popular as feed supplements in the aquaculture sector (Chu 2012; Priyadarshani and Rath 2012), thanks to the functional properties of their pigments and bioactive compounds. In addition, they may have a high content of proteins (30–70%), lipid (10–20%), and essential fatty acids (Becker 2007; Nasir et al. 2015; Shah et al. 2018; Cardinaletti et al. 2018). The main microalgal cultured genera are *Chlorella*, *Nannochloropsis*, *Scenedesmus*, *Arthrospira*, *Tisochrysis*, and *Tetraselmis* (Sirakov et al. 2015; Bleakley and Hayes 2017), thanks to their nutritional and health properties and consolidated cultivation technology. Recently, Castro et al. (2020) have proved that the inclusion up to 15 % of *Nannochloropsis* sp. in diets for European sea bass has decreasing effects on the liver and intestinal antioxidant activity, while Abdelghany et al. (2020) have demonstrated that dietary *N. oculata* significantly improves growth parameters and resistance to pathogens such as *Aeromonas veronii* in Nile tilapia (*O. niloticus*).

However, the use of microalgae as an ingredient of aquafeeds may imply a few drawbacks such their high costs. In the recent decades, many biotechnological processes have emerged that are based on microalgal cultures and significant both from the environmental and industrial point of view. Such processes include biogas enrichment and purification; wastewater treatment (Quijano et al. 2017); CO_2_, NOx, and SOx removal from flue gas (Yen et al. 2015); and recovery of added-value products such as pigments, nutraceuticals, fertilizers, and biofuels (Bahr et al. 2013). The traditional processes for wastewater treatment are very expensive due to the chemical additives required during each phase. However, the cost could be minimized by using the microalgae biomass obtained by this technology as a feed for aquaculture. Some researchers have thus studied the potential value of multiple applications of microalgae to contribute to a circular economy approach (Valente et al. 2019) through their use in wastewater treatment (Velichkova et al. 2014; Nasir et al. 2015) and the sustainable production of biofuels (Rawat et al. 2011; Oliveira et al. 2020).

Numerous studies have been conducted on the characteristics of the microalgae obtained by a phycoremediation process (Yaakob et al. 2014; Nasir et al. 2015; Badr et al. 2019; Apandi et al. 2019; Michelon et al. 2021). Phycoremediation is a biotechnological process to remove contaminants from wastewater and is considered a simpler method than the conventional one (Raskin et al. 1997; Atiku et al. 2016). Microalgae have already been used to remove inorganic molecules and improve water quality (Ruiz-Martinez et al. 2012). Moreover, wastewater from the fish farm and the fresh market has also been used as a medium for a non-axenic microalgae culture (Apandi et al. 2019; Andreotti et al. 2017; Michels et al. 2014).

In general, wastewater contains a high level of nutrients (nitrogen, phosphorus, and carbon) and organic matter, which act as elements to support microalgae biomass (Riaño et al. 2016); nitrogen availability has been shown to improve biomass production (Maizatul et al. 2017), thus modulating their nutritional value. Michels et al. (2014) used the wastewater obtained from a fish farm as a culture medium for the non-axenic production of *Tetraselmis suecica* biomass that in turn was used in juvenile shellfish culture resulting in increased productivity and constant quality in the hatchery phase. Several processes at pilot or industrial scale are actually based on non-axenic microalgae cultures from wastewater treatment, biogas purification/upgrading, or flue gas treatment. Moreover, recent studies have proved that microalgae biomass and composition, such as lipid composition, can be adjusted under physiological stress conditions, namely nitrogen depletion with increased salinity and/or increased salinity with temperature shock (Markou et al. 2016; Anitha et al. 2018). In non-axenic production processes, both microalgal and bacterial communities play key roles (Coronado-Apodaca et al. 2019), and the combination of different microalgal species can provide a balanced diet and improve animal growth and welfare (Spolaore et al. 2006; Cardinaletti et al. 2018). Cultivating microalgae in a high-rate algal pond (HRAP) system is a simple and economic way to produce valuable biomass to be included in aquafeeds. It provides fish farm wastewater treatment allowing the re-use of water for aquaculture while providing free nutrients for microalgae biomass production (Craggs et al. 2014). In addition, the technological treatment of microalgae biomass could also represent an important source of proteins, n-3 rich lipids, antioxidants, and natural bioproducts.

In this context, this study aimed to test the effects of dietary inclusion of a microalgae consortium grown in a HRAP system on zootechnical performance, morphometric indices, and dietary nutrient digestibility, as well as on the morphology and functionality of the digestive system of European sea bass. A dietary treatment including the commercial mono-cultured microalgae *Nannochloropsis* sp. was used for comparison, based on its nutritional properties and, in particular, n-3 PUFA content.

## Materials and methods

### Microalgae consortium production and characterization

**Fig. 1:**
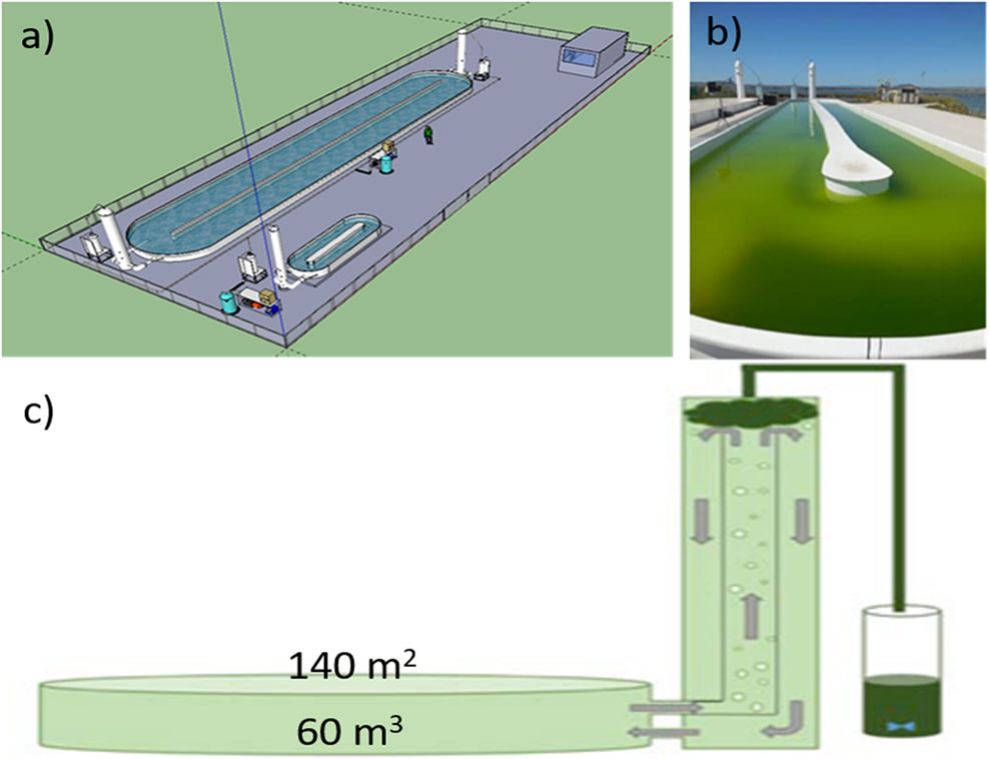
High-rate algal pond. (a) general view; (b) picture of the consortium culture; (c) crossing section view with COLDEP® column and harvest collector

The trial was conducted at the Ifremer experimental station in Palavas les Flots, France. The microalgae consortium was cultivated in a conventional oval-shaped raceway HRAP. Water mixing in the HRAP (140 m^2^ and 60 m^3^) was maintained at 0.2 m^3^/s using a vacuum airlift column developed and patented by COLDEP® (Barrut et al. 2012; Barrut et al. 2013). The column was connected to the HRAP and consisted of a central tube, the top of which was hermetically closed and connected to a vacuum pump. Water was raised to the top of the central tube with a vacuum and allowed to flow over the central tube so that it could be returned to the HRAP (Fig. 1). The raceway was initially filled with natural marine water filtered at 30 μm and supplied with an effluent profile corresponding to European sea bass (*Dicentrarchus labrax*) breeding tanks providing 80 g N/day and 30 g P/day for 75 days. The total biomass profile consisted of 2000 fish of 80±2.3 g (average body weight) fed with a fixed daily rate (1.2% of the biomass). The experimental natural consortium of marine microalgae was grown under a natural irradiance directed by the local weather at 43° 31′ 59.98″ N, 3° 55′ 59.99″ E in autumn 2017 in France on the western Mediterranean coast. CO_2_ addition flow was adjusted by an automatic pH detection device which was adjusted to photosynthetic demand based on pH level monitoring (Galès et al. 2020).

Chlorophyll a concentrations were measured (Lorenzen 1967) twice a week during the exponential period of growth, corresponding to the sample collection period. The data showed an increasing concentration from 0.8 to 3.5 mg/L of chlorophyll a. Culture productivity calculated on the sampling period was 2.53 g/m^2^/day. Algal consortium biomass was weekly sampled until 5 kg of dried biomass was obtained. Natural algae concentration was pre-concentrated using COLDEP® column (around 10- to 20-fold depending on the culture stage) and centrifuged using a plate divider Alfa Laval “Clara15” to obtain a paste featuring an approximate 7% dryness. Residual water was extracted by freeze-drying, and the final product was ground to obtain a meal mesh comparable to the industrial fish meal. The dried consortium biomass (5 kg) was defined in terms of nutrients before being used at graded levels in formulated feeds satisfying the European sea bass nutritional requirements (Peres and Oliva-Teles 1999a).

The species composition of the consortium was determined using 18S rRNA gene analysis. For each experimental run, 10mL samples were filtered through 0.2μm membranes (PALL ALL Supor® 200 PES), the membranes being stored at −20 °C for subsequent DNA extractions. The DNA was extracted using DNeasy PowerWater Kit (Qiagen) according to the manufacturer’s instructions. The V4 region of the 18S rRNA gene was amplified over 30 amplification cycles at an annealing temperature of 65°C, with forward and reverse primers (5′-CTTTCCCTAACGACGCTCTTCCGAT CTGCGGTAATTCCAGCTCCAA-3′ and 5′-GGAGTTCAGACGTGTGCTCTTCCGATCTTTGGCAAATGCTTTCG C-3′, respectively). The resulting products were purified and loaded onto an Illumina MiSeq cartridge for sequencing, paired with 300bp reads following the manufacturer’s instructions (v3 chemistry). Sequencing and library preparation steps were carried out at the Genotoul Lifescience Network Genome and Transcriptome Core Facility in Toulouse, France (get.genotoul.fr). A modified version of the standard operation procedure for MiSeq data (Kozich et al. 2013) in Mothur version 1.35.0 (Schloss et al. 2009) was used for alignment and taxonomic outline. Mothur was also used to identify representative sequences of operational taxonomic units (OTUs).

### Test ingredients and diets

Four diets were formulated to be isoproteic (48.5%±0.8) and isolipidic (18.3%±0.5). As a control diet (C), a formulation was used that simulated a commercial diet containing fish meal and oil and vegetable-derived protein mix, including solvent-extracted soybean meal, pea protein concentrates, and wheat meal. The microalgae consortium was included to partially replace the vegetable-derived protein mix in diet MC10 (10% replacement) and diet MC20 (20% replacement) (Table 1).

**Table 1:** Ingredients (g/kg) and proximate composition, phosphorus and energy content of the test diets

	CTRL	MC10	MC20	N10
Ingredients				
Fishmeal Chile prime	25.25	25.25	25.25	25.25
Vegetable mix§	37.87	35.05	34.03	36.02
Wheat gluten meal	4.04	5.05	5.05	0.00
Wheat meal	17.17	9.09	0.00	14.74
Fish oil	13.94	13.73	13.73	12.12
Microalgal consortium	0.00	10.10	20.20	0.00
*Nannochloropsis* sp.#	0.00	0.00	0.00	10.10
Min. and Vit. supplement$	1.00	1.00	1.00	1.00
Yttrium oxide	0.02	0.02	0.02	0.02
Binder	0.20	0.20	0.20	0.20
l-Methionine	0.50	0.50	0.50	0.50
Chemical composition				
Dry matter (%)	96.94	96.87	97.17	97.12
Protein (% DM)	49.17	49.26	47.61	48.24
Lipids (% DM)	18.14	17.84	18.18	19.12
Ash (% DM)	7.84	12.80	18.33	10.29
Phosphorus (% DM)	1.03	1.07	1.11	1.13
Gross energy (MJ/kg)	23.10	22.20	21.20	22.80

§Vegetable mix: including soy protein concentrate, pea protein concentrate, solvent extracted soybean meal in a 4:1:4 ratio

#*Nannochloropsis* from GREENSEA, Meze-Fr

$Mineral supplement composition (% mix): CaHPO_4_*2H_2_O, 78.9; MgO, 2.725 g; KCl, 0.005; NaCl, 17.65; FeCO3, 0.335; ZnSO_4_*H_2_O, 0.197; MnSO_4_*H_2_O, 0.094; CuSO_4_*5H_2_O, 0.027; Na_2_SeO_3_, 0.067

Vitamin supplement composition (% mix): thiamine HCL Vit B1, 0.16; riboflavin, Vit B2, 0.39; pyridoxine HCL Vit B6, 0.21; cyanocobalamine B12, 0.21; niacin Vit PP, 2.12; calcium pantotenate, 0.63; folic acid, 0.10; biotin Vit H, 1.05; myoinositol, 3.15; stay C Roche, 4.51; tocoferol Vit E, 3.15; menadione Vit K3, 0.24; Vit A (2500 UI/kg diet) 0.026; Vit D3 (2400 UI/kg diet) 0.05; choline chloride, 83.99

A diet (N10) including 10% of commercial *Nannochloropsis* sp. dry biomass was used for comparison. *Nannochloropsis* sp. cells were cultivated in a photobioreactor using industrial chemical fertilizer. The spray-dried *Nannochloropsis* biomass was provided by an industrial algae farm (GREENSEA, Meze-Fr).

Diets were supplemented with l-methionine so that the sulfur amino acid level met the requirements of the European sea bass (Tulli et al. 2010). Yttrium oxide (20 mg/100 g diet) was added as an indigestible marker to assess nutrients and energy digestibility of the test diets. The diets were manufactured by INRA in Donzacq (F) as standardized 2mm pellets. The ingredients and proximate composition of the experimental diets are shown in Table 1.

### Experimental animals and feeding trial

Six hundred thirty-six European sea bass (*Dicentrarchus labrax*) juveniles were purchased from a commercial hatchery (Poissons du Soleil, Balaruc les-Bains, France).

After 3 weeks of acclimation to the experimental conditions, the fish (average initial body weight 18.0 ± 0.28 g) were randomly allotted among 12 cylindrical tanks, featuring a volume of 1 m^3^ each (53 fish per tank) and equipped with a collection tube for feces and uneaten pellets in a recirculation aquaculture system (RAS), thus ensuring optimal water conditions for European sea bass (Table 2), and were fed a commercial diet. At the beginning of the feeding trial, the fish were individually implanted with a microchip (Biomark Inc., ID, USA) under moderate anesthesia (90 ppm benzocaine) (Topic Popovic et al. 2012).

**Table 2:** Average and range values of physico-chemical water parameters over 75 days

Parameter	Average	Min.	Max.
Salinity (g/L)	35.3	27.8	39.1
Temperature (°C)	22.8	19.7	23.2
pH	7.1	6.5	7.6
Dissolved oxygen (mg/L)	7.6	5.6	6.7
N-NH_3_ (ppm)	0.3	0.1	0.8
N-NO_2_ (ppm)	0.0	0.0	0.0
N-NO_3_ (ppm)	1.4	0.4	3.3
P-PO_4_ (ppm)	0.1	0.0	0.2

The fish were assigned to fish groups/tanks according to a completely random design with diets as the main factor and three replicates per treatment and hand-fed the experimental diets starting on 28^th^ March 2018 over 75 days to apparent satiation in 3 daily meals from 8 am to 2 pm. The fish were group-weighed every 4 weeks and at the end of the feeding trial, under moderate anesthesia after 40hours fasting. Relative feed intake (RFI= feed intake/[(Initial body weight +Final body weight) × 0.5 × days]), specific growth rate (SGR= 100 × (ln Final body weight − ln Initial Body Weight)/days), feed conversion ratio (FCR= feed intake/weight gain), protein efficiency ratio (PER= weight gain/protein intake), and gross protein retention (GPR= 100 × [(final body protein content-initial protein content)/protein intake]) were calculated.

At the end of the feeding trial, after 40hours fasting, 3 fish per tank (9 fish per dietary treatment) were sacrificed with a lethal solution of benzocaine (200 ppm; Vignet et al. 2014). Individual weight and length and viscera, liver, and mesenteric fat weight were recorded. The intestinal tract was excised for histological and physiological evaluations: Fulton’s condition index (K= body weight/standard length^3^), viscerosomatic index (VSI= 100 × viscera weight/body weight), hepatosomatic index (HSI= 100 × liver weight/body weight), mesenteric fat index (MFI= 100 × mesenteric fat/body weight), and carcass yield = 100 × carcass weight/body weight were calculated.

### Diet digestibility evaluation

To evaluate the in vivo nutrient digestibility of the test diets, fish feces were daily collected from each tank during the last 3 weeks of the feeding trial and preserved at −20°C until used. Fecal biomass was centrifuged (10 min at 3000×*g* at 4°C), freeze-dried, and stored (−20 °C) until analyzed. Feed and feces were analyzed for dry matter (AOAC 934.01), protein (AOAC 2001.11), lipids (AOAC 2003.05), and energy (ISO 9831-1998) (IKA – C7000) content. Yttrium concentration in feed and feces was determined by inductively coupled plasma mass spectrometry (ICP-MS) according to Carignan et al. (2001). Apparent digestibility coefficients (ADCs) of dry matter, protein, lipid, and energy of the diets were calculated according to the following formula:

$$ \mathrm{ADC}=1\hbox{--} \left[\left(\frac{F}{D}\right)\ast \left(\frac{Di}{Fi}\right)\right] $$

where D = % of the nutrient or kJ/g of the energy in the diet; F = % of the nutrient or kJ/g of the energy in the feces; Di = % Y in the diet; and Fi = % Y in the feces (Cho et al. 1982).

### Gut histology

**Fig. 2:**
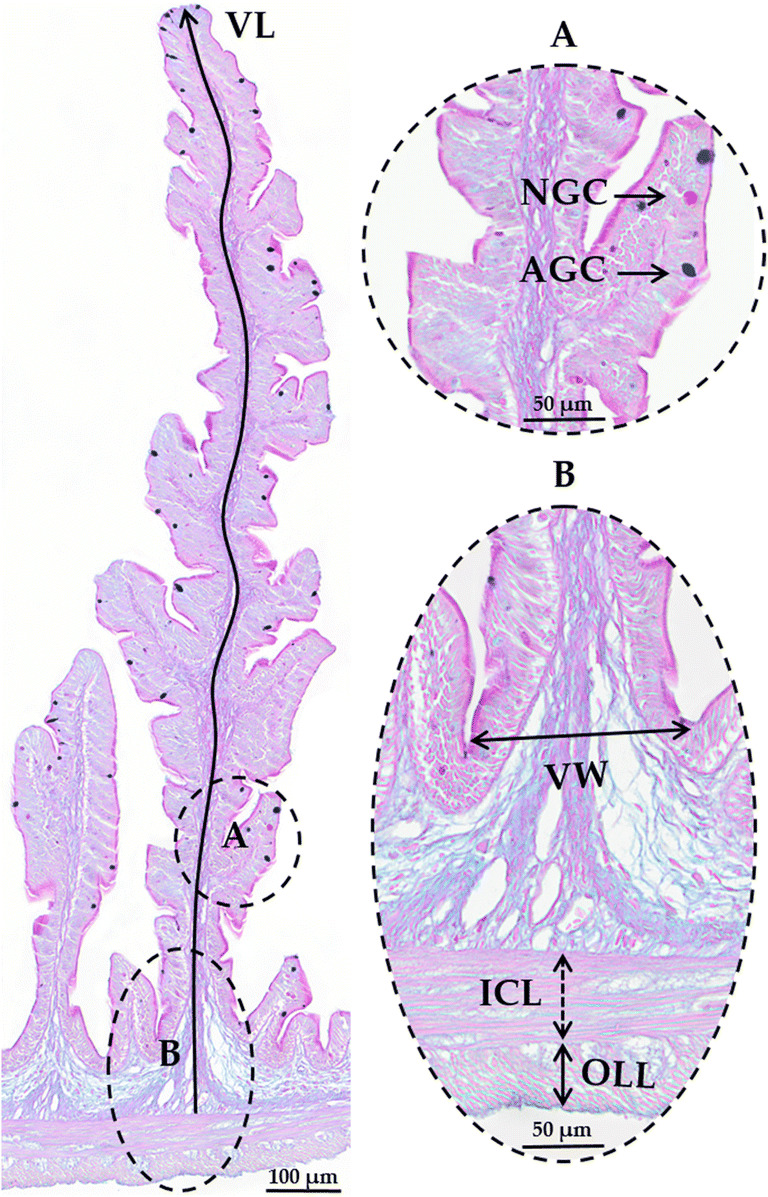
Anterior intestine histological sections (Alcian Blue/PAS staining, pH = 2.5) of European sea bass. Villus length (VL), muscularis externa (outer longitudinal layer OLL and inner circular layer ICL); villus width (VW), Goblet cells (acid AGC and neutral NGC)

Two fish from each tank were used for histologic analyses. Fish gut was dissected, and proximal intestine samples were collected from below the pyloric caeca (0.5cm fragment). Samples were fixed in 4% neutral-buffered formaldehyde and embedded in paraffin. Cross sections of each sample were cut (3 μm thick) in a semi-automated rotary microtome (Leica RM 2245). Slides were then dewaxed and stained with specific Alcian Blue/PAS (pH=2.5). Micrographs of each section were obtained through slides scanning using a VS120 Virtual Slide Microscope (20× magnification). On each intestinal section, the following parameters were measured in two sections of each sample by using imaging software Olympus cellSens Dimension Desktop: cross-sectional area; muscularis externa thickness (inner circular and outer longitudinal muscle layers); fold length and width and goblet cell presence, as previously described (Batista et al. 2020a, b) (Fig. 2). Briefly, the muscularis externa was measured in eight points of each cross section, and the mean value was considered; the eight highest folds in each section were selected to measure their length and width. Goblet cells (mucus-producing cells) were counted in the eight selected folds (blue and magenta cells), and the average number of goblet cells per fold was determined.

### The activity of the brush border membrane (BBM) enzymes

One fish per tank was used to obtain the digestive tract that was divided into pyloric *caeca* (PC), proximal intestine (P, the section from below the PC to the increase in diameter indicating the start of the distal intestine), and distal intestine (D, the terminal part of the intestine with a larger diameter, reaching the anus). When necessary, the remaining feed residues were gently squeezed out. Tissue samples were lightly blotted with absorbent paper, put in individual plastic tubes, and stored at −20°C until the analysis of the BBM enzyme activity was performed. The extraction of the BBM enzymes and the analysis of maltase, sucrase-isomaltase (SI), γ-glutamyl transpeptidase (γ-GT), and alkaline phosphatase (ALP) were carried out as reported by Messina et al. (2019). One unit (U) of enzyme activity corresponded to the amount of enzyme that transforms or hydrolyses 1 μmol of substrate/mL^/^min. The specific enzymatic activity was calculated as U = μm/min/mg of supernatant protein for maltase and sucrase-isomaltase and mU for ALP and γ-GT.

The amount of total protein in the supernatant was determined according to Bradford et al. (1976) by using Bradford reagent (Sigma-Aldrich, Milan, Italy) and bovine serum albumin (Sigma-Aldrich, Milan, Italy) as a standard.

### Statistical analysis

Data are expressed as average ± standard deviation. Zootechnical and digestibility data were analyzed by one-way ANOVA to test statistical significance within the main factor. BBM enzyme activity data were analyzed by a two-way ANOVA test, considering the dietary treatment and the intestinal section as main factors. If appropriate, Duncan’s post hoc test was applied at a significant level of 95%. IBM-SPSS statistical package (release 17.0) was used to carry out data analysis.

## Results

### Marine consortium characterization

The consortium biomolecular characterization identified 34 assignments with 6 dominant algal species (Table 3). The main species of the natural consortium were as follows: *Oocystis* sp*.*, *Chlorella stigmatophora*, *Tetraselmis* sp*.* Depending on the open pond culture cycle and the season, *Isochrysis* sp*.* and *Phaeodactilum tricornutum* were observed in the minority.

**Table 3:** Taxonomic composition of the marine consortium

	Quantity (%)	Properties	Reference
Algae			
*Oocystis* sp.	80.32	High EPA	Anthony and Stuart 2015
*Tetraselmis* sp.	6.06	High EPA and ARA	Vizcaíno et al., 2016
*Chlorella stigmaphora*	2.06	High EPA	Anthony and Stuart 2015
*Chlamydomonas* sp.	1.22	Mineral (boric acid and calcium)	Kliphuis et al. 2012
*Nannochloropsis gaditana*	0.06	15% EPA	Anthony and Stuart 2015
*Nannochloris* sp.	0.06	35% EPA	Anthony and Stuart 2015
Others			
Rotifera	5.84	Monounsaturated fatty acid	Awaiss et al. 1992
Lacrymariidae (ciliated)	2.09	x	
Cinerochilidae (Phylasterides)	2.07	x	
Chytridiomycotina (Chytridiomycota)	0.18	x	
Strombidiidae (ciliated)	0.02	x	
*Isochrysis*	Traces	x	
*Phaeodactylum tricornutum*	Traces	x	

The chemical composition of the dried consortium biomass is shown in Table 4. The consortium was characterized by 2.8% nitrogen and 3.2% total lipid. Oleic (16.8% FAMEs) and linolenic (12.4% FAMEs) acids were the main fatty acids. The free amino acid fraction was dominated by proline, alanine, arginine, and glutamate (42.0, 21.3, 16.0, 16.5 nmol/mg, respectively). Natrium, iron, and boron were the most abundant elements in the mineral fraction.

**Table 4:** Major chemical characteristics of the dried marine consortium biomass

Nitrogen (%)	2.8	
Lipid (%)	3.2	
Gross energy (kJ/g)	9.0	
Fatty acids (% FAMEs)		
18:1 n-9	16.8	
16:3 n-3	4.3	
16:4n-3	1.9	
18:3n-3	12.4	
20:3n-3	1.1	
20:4n-3	1.1	
20:5n-3	9.1	
22:5n-3	0.2	
SFA	24.0	
MUFA	32.0	
PUFA	42.0	
n-3	33.0	
n-6	8.0	
Amino acids (nmol/mg)	Free	Hydrolysed
Arginine	16.0	66.0
Histidine	0.0	15.0
Lysine	3.0	47.0
Threonine	1.6	50.0
Isoleucine	1.0	40.0
Leucine	1.0	40.0
Valine	2.0	64.0
Methionine	1.0	14.0
Phenylalanine	1.0	37.0
Tryptophan	0.0	0.0
Alanine	21.3	111.0
Tyrosine	1.0	20.0
Aspartate	1.2	82.0
Glutamate	16.5	92.0
Glycine	4.3	101.0
Serine	3.3	51.0
Proline	42.0	88.0
Minerals (μg/g)		
B	55.1	
Ca	6.4	
Cd	0.4	
Co	2.9	
Cr	1.1	
Cu	5.2	
Fe	84.8	
K	10.4	
Mg	16.7	
Mn	9.6	
Na	120.5	
Ni	0.3	
P	5.2	
Zn	8.0	
Carbohydrates (%)		
Galactose	1.9	
Glucose	6.6	
Mannose	2.2	
Xylose	1.9	
Fucose	<0.5	
Rhamnose	7.5	
Gluconic acid	1.2	

### Fish growth performance

During the experimental period, the fish easily accepted the experimental diets, and mortality was negligible. Growth performance, RFI, FCR, and PER of the European sea bass juveniles fed with the experimental diets over 75 days are shown in Table 5. The fish fed with diet MC10 exhibited a significantly higher final body weight as compared to those fed with the control diet (64.9 vs 61.0 g; P<0.05), while the SGR value in MC10, though being the highest, did not reach statistical significance (P = 0.066). The fish fed with diet MC20 exhibited the highest relative feed intake (18.4 g/kg ABW/day), a significantly different value from the RFI of the C group that showed the lowest one (16.7 g/kg ABW/day) (P<0.05). Feeding diet MC20 also resulted in a significant increase in FCR values as compared to the other dietary treatments (1.25 vs 1.15 respectively, P = 0.0052). On the contrary, PER was significantly lowered by the microalgae inclusion in the MC20 group compared to diet C and Nannochloropsis-including diet (1.73 vs 1.83, P<0.05). GPR was not affected by the experimental diets.

**Table 5:** Zootechnical performance of European sea bass fed the experimental diets over 75 days

	CTRL	MC10	MC20	N10	P
Mortality (%)	1	0	1	0	
Initial body weight (g)	18.00 ± 1.10	18.50 ± 0.90	18.50 ± 1.20	18.40 ± 1.00	0.088
Final body weight (g)	61.0 ± 11.00b	64.9 ± 13.40a	63.4 ± 12.80ab	63.70 ± 12.70ab	0.024
RFI (g/kg ABW/day)^1^	16.70 ± 0.60b	17.4 ± 0.10ab	18.4 ± 0.30a	17.30 ± 0.30ab	0.018
SGR (%)^2^	1.65 ± 0.02	1.70 ± 0.01	1.66 ± 0.03	1.68 ± 0.02	0.066
FCR^3^	1.15 ± 0.03b	1.16 ± 0.01b	1.25 ± 0.03a	1.16 ± 0.03b	0.005
PER (%)^4^	1.82 ± 0.04a	1.80 ± 0.02ab	1.73 ± 0.04b	1.84 ± 0.05a	0.007
GPR (%)^5^	32.09 ± 1.75	31.33 ± 0.87	30.05 ± 1.76	29.10 ± 1.28	0.075

Data are presented as mean ± standard deviation; values with different letters on the same row are significantly different (P < 0.05), n= 3

^1^$$ RFI\ \left( Relative\ Feed\ Intake\right)=\frac{\mathrm{feed}\ \mathrm{intake}\ }{\left(\mathrm{Initial}\ \mathrm{Body}\ \mathrm{Weight}\kern0.5em +\mathrm{Final}\ \mathrm{Body}\ \mathrm{Weight}\right)\times 0.5\times \mathrm{days}} $$

^2^$$ SGR\ \left( Specific\ Growth\ Rate\right)=100\times \frac{\ln \mathrm{Final}\ \mathrm{Body}\ \mathrm{Weight}-\ln \mathrm{Initial}\ \mathrm{Body}\ \mathrm{Weight}}{\mathrm{days}} $$

^3^$$ FCR\ \left( Feed\ Conversion\ Ratio\right)=\frac{\mathrm{feed}\ \mathrm{intake}}{\mathrm{weight}\ \mathrm{gain}} $$

^4^$$ PER\ \left( Protein\ Efficiency\ Ratio\right)\kern0.5em =\frac{\mathrm{weight}\ \mathrm{gain}}{\ \mathrm{protein}\ \mathrm{intake}} $$

^5^$$ GPR\ \left( Gross\ Protein\ Retention\right)=100\times \left[\frac{\left(\mathrm{Final}\ \mathrm{Body}\ \mathrm{protein}\ \mathrm{content}-\mathrm{Initial}\ \mathrm{Body}\ \mathrm{protein}\ \mathrm{content}\right)}{\ \mathrm{protein}\ \mathrm{intake}}\right] $$

The biometric morphometric index values calculated at the end of the trial on European sea bass did not reveal any significant effects of the experimental diets (Table 6).

**Table 6:** Biometric morphometric indices of European sea bass fed the experimental diets over 75 days

Biometric index	CTRL	MC10	MC20	N10	P value
K^1^	1.72 ± 0.11	1.73 ± 0.11	1.70 ± 0.10	1.73 ± 0.12	0.108
HSI (%)^2^	1.18 ± 0.31	1.03 ± 0.27	1.02 ± 0.28	1.27 ± 0.70	0.539
VSI (%)^2^	10.0 ± 1.20	9.54 ± 1.00	9.64 ± 1.90	11.0 ± 1.90	0.162
MFI (%)^2^	5.93 ± 1.12	5.18 ± 0.90	4.85 ± 1.13	5.77 ± 2.13	0.344
Carcass yield (%)^2^	82.89 ± 2.28	84.26 ± 1.90	83.75 ± 2.29	81.19 ± 3.76	0.101

Data are presented as mean ± SD; values with different letters in the same row are significantly different (P < 0.05) n=9

^1^$$ K\ \left( Fulton's\  Condition\ Index\right)=\frac{\mathrm{body}\ \mathrm{weight}}{\mathrm{std}\ {\mathrm{lenght}}^3} $$

^2^$$ HSI, VSI, MFI, Carcass\ Yield=100\times \frac{\ \mathrm{weight}\ \mathrm{of}\ \mathrm{liver},\mathrm{viscera},\mathrm{mesenteric}\ \mathrm{fat},\mathrm{carcass}\ }{\mathrm{body}\ \mathrm{weight}} $$

### Diet digestibility

The dry matter, protein, lipid, and energy ADCs of the experimental diets are shown in Table 7. Diet N, including 10% of *Nannochloropsis* sp., resulted in ADC values similar to the ones observed in the reference C diet (74.2, 92.2, 85.4, and 85.8 %, respectively for dry matter, protein, lipid, and energy). On the contrary, increasing the dietary inclusion of the marine consortium resulted in a significant decrease in dry matter, protein, lipid, and energy ADCs as observed in MC10 (65.9, 90.0, 82.8, 80.1 % respectively) and MC20 (57.7, 85.2, 84.9, 75.2 % respectively) diets (P<0.05).

**Table 7:** Nutrient and energy apparent digestibility coefficients (%) of the experimental diets

	CTRL	MC10	MC20	N10	P value
Dry matter	76.7 ± 1.0a	65.9 ± 2.0bc	57.7 ± 6.2c	74.2 ± 1.1ab	0.000
Protein	92.7 ± 0.3a	90.0 ± 0.5b	85.2 ± 1.2c	92.2 ± 0.1a	0.000
Lipid	86.8 ± 0.2a	82.8 ± 0.8b	84.9 ± 2.3ab	85.4 ± 0.3ab	0.023
Energy	87.4 ± 0.9a	80.1 ± 1.3b	75.2 ± 3.4c	85.8 ± 0.8a	0.000

Data are presented as mean ± SD; values with different letters in the same row are significantly different (P < 0.05) n=3

### Intestine morphology

**Fig. 3:**
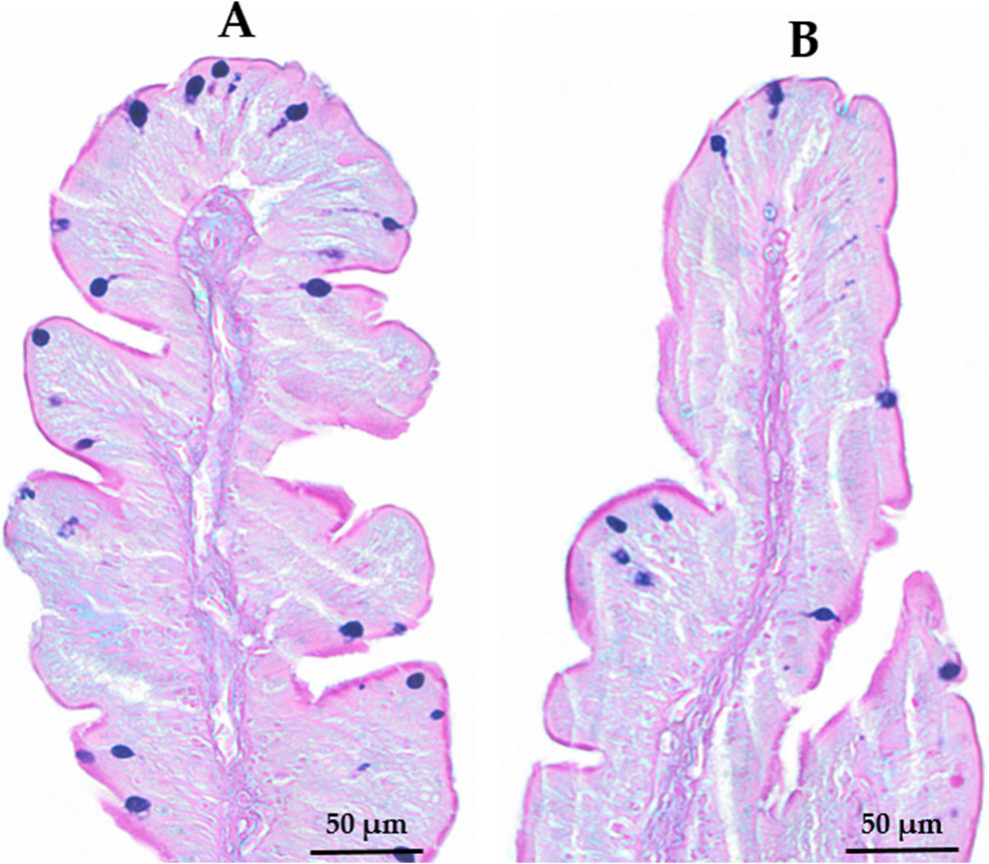
Anterior intestine histological sections (Alcian Blue/PAS staining, pH = 2.5) of European sea bass at the end of 75-day feeding trial. Blue points represent the acid goblet cells. A, CTRL diet and B, MC20 diet

The fish fed with the experimental diets did not exhibit major alterations in intestinal morphology, as shown in Table 8. Most of the traits considered did not vary significantly among the dietary treatments (P>0.05), and the intestine from all sampled fish showed a well-preserved morphology. A significant reduction in the total number of acid goblet cells per fold was registered in the fish fed with the highest consortium dietary inclusion level as compared to those fed with the control diet (36.77 vs 64.29 n. GC/fold respectively for MC20 and C, P<0.05) (Fig. 3).

**Table 8:** Intestinal morphology of European sea bass fed the experimental diets over 75 days

	CTRL	MC10	MC20	N10	P value
Cross-sectional area (mm^2^)	11.2 ± 2.97	11 ± 4.41	9.5 ± 2.29	10.2 ± 1.62	0.761
Villus length (μm)	1414.7 ± 256.7	1416.5 ± 368.5	1327.6 ± 207.9	1240.0 ± 126.6	0.592
Villus width (μm)	226.9 ± 34.76	225.1 ± 32.41	222.3 ± 29.50	225.9 ± 14.85	0.993
Muscularis externa (μm)	131.7 ± 20.45	104.4 ± 21.69	106.5 ± 30.88	129.3 ± 17.68	0.103
Inner circular layer (μm)	85.1 ± 13.23	67.4 ± 14.23	65.6 ± 18.58	81.4 ±10.69	0.069
Outer longitudinal layer (μm)	46.6 ± 8.26	37 ± 7.83	40.9 ± 13.23	47.9 ± 9.40	0.226
Goblet cells (no. GC/fold)	88.8 ± 16.94	67.2 ± 20.55	56.7 ± 13.05	82.2 ± 29.64	0.061
Acid GC (no. GC/fold)	64.3 ± 13.46a	46.4 ± 8.14ab	36.8 ± 6.26b	54.7 ± 17.44ab	0.006
Neutral GC (no. GC/fold)	24.6 ± 15.54	20.8 ± 14.92	19.9 ± 8.13	27.6 ± 15.21	0.760

Data are presented as mean ± SD; values with different letters in the same row are significantly different (P < 0.05), n=6

### The activity of the intestinal brush border membrane enzymes

**Fig. 4:**
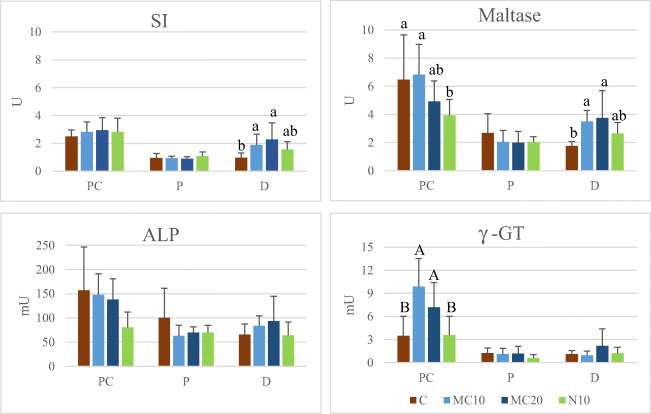
Specific activity of SI (sucrase-isomaltase), maltase, ALP (alkaline phosphatase), and γ-GT (gamma glutamil transpeptidase) in PC (pyloric caeca), P (proximal intestine), and D (distal intestine) of European sea bass fed the experimental diets over 75 days. Data are presented as means ± SD (n=3). Different letters indicate significant differences among the treatment diets (lower case superscript P < 0.05, capital superscript P < 0.001).

The activity of maltase, SI, -γGT, and ALP varied depending on the intestinal tracts (Fig. 4). The PC was the major site of activity for all enzymes studied. In this tract, the diet containing 10% of *Nannochloropsis* sp. resulted in a significant decrease of the maltase activity as compared to the control diet (3.83 vs 6.41 U, P<0.05).

In the distal portion of the intestine, the activity of SI and maltase showed a similar pattern and their activity resulted significantly enhanced in the fish fed with the consortium-including diets as compared to diet C (2.05 vs 0.96 U and 3.63 vs 1.76, respectively, P<0.05).

The dietary treatment considerably affected the activity of γ-GT in the PC. The highest value was observed in the fish fed with the MC10 diet (P=0.001). The activity of ALP was not affected by the dietary treatments (P > 0.05).

The effect of the two main factors (diet and intestinal tract) was tested on the brush border membrane enzyme activity of the European sea bass fed with the experimental diets. The two-way ANOVA results are summarized in Table 9. A significant interaction between the main factors was revealed for maltase and γ-GT.

**Table 9:** ANOVA of the main factors which affect the activity of the BBM enzymes

	Diet	Tract	Diet × Tract
Maltase	*	**	**
Sucrase	*	**	NS
ALP	*	**	NS
γ-GT	**	*	**

*P < 0.05; **P < 0.001

## Discussion

The interest in the use of microalgae dry biomass in aquafeeds is recent; several studies already tested the effects of their dietary inclusion in the in vivo trials on different fish species (Hussein et al. 2013; Eryalçın and Yıldız 2015; Haas et al. 2016; Kissinger et al. 2016; Vizcaíno et al. 2016a; Sarker et al. 2020).

Different microalgae species such as *Gracilaria gracilis*, *Nannochloropsis oceanica*, *Tisochrisis lutea*, and *Tetraselmis suecica* have been used as partial replacement of fish meal in diets for European sea bass with no adverse effects on zootechnical performance and intestinal physiology (Cardinaletti et al. 2018; Messina et al. 2019; Valente et al. 2019; Batista et al. 2020a). *Isochrysis* sp. has also been proposed as a source of n-3 PUFA in partial substitution of fish meal in diets for European sea bass without any effects on feed intake and zootechnical performance (Tibaldi et al. 2015). Similar results in the same fish species have been obtained by Haas et al. (2016) when part of fish oil was substituted by *Pavlova viridis* and *Nannochloropsis* sp. Moreover, treatment with microalgae in this trial did not affect the histological aspect of the liver and intestine. Studies carried out on rainbow trout have demonstrated the effectiveness of microalgae when included at low levels in the diets. Sarker et al. (2020) demonstrated that *Isochrysis* sp. and *Schizochytrium* sp. are possible candidates for DHA supplementation in rainbow trout diet formulations, while Sheikhzadeh et al. (2012) showed that dietary *Haematococcus pluvialis* might enhance the antioxidant system when added at 0.3 %.

However, contrary to the vast majority of studies with microbial biomass, the marine consortium tested in the present study consisted of a certain number of different organisms, namely *Oocystis* sp. (80%), *Tetraselmis* sp. (6%), *Chlorella stigmaphora*, *Chlamydomonas* sp., *Nannochloropsis gaditana*, *Nannochloris* sp., Rotifera, Lacrymariidae (ciliated), Cinerochilidae (phylasterides), Chytridiomycotina (Chytridiomycota), and Strombidiidae (ciliated). The analysis method cannot precisely define the *Oocystis* species, but whereas *Oocystis* sp. is generally associated with a freshwater genus of green microalgae, oceanic strains can be found in marine or brackish water such as *Oocystis submarina* (Śliwińska-Wilczewska and Latała 2018) or *Oocystis borgei* which could inhibit harmful microalgae by expressing allopathic effects (Wang et al. 2020). *Oocystis heteromucosa* belongs to strains of marine algae consortium found in marine aquaculture pond wastewater with a high ammonia tolerance (Katayama et al. 2020). In a HRAP, algae predators such as rotifers can have a negative effect on the consortium growth to the point of cultural annihilation. That was not the case in our experiment: we hypothesize that continuous CO_2_ delivery linked to the photosynthetic demand maintains a pH value around 7, which could be uncomfortable for organism reproduction adapted to marine pH at 8.2. In addition, the culture did not collapse, probably because of the high culture volume and dynamic algae growth. Previous experiments showed that the algae cells’ reproduction rate has to be higher than the total grazers’ reproduction rate in order to keep a culture alive (Strom and Morello 1998). That was the case in the exponential culture growth phase, which is the sample period for biomass extraction. Algae predators also sequester compounds of some interest from algae ingestion and their tolerated presence in the culture contributes to final powder value.

The use of a non-axenic culture of a blend of *Tetraselmis suecica*, *Isochrysis galbana*, and *Dunaliella tertiolecta* has been evaluated in its remediation potential for the nutrient assimilation of fish farm wastewater (Andreotti et al. 2017). Dallaire et al. (2007) have previously described the effect on trout fry of the dietary inclusion of a freshwater photosynthetic microorganism consortium (mainly *Scenedesmus* sp. and *Chlamydomonas* sp.) derived from the sedimentation pond of a fish farm. The results showed that a maximum of 12.5% of consortium could be included in the feed formulation without affecting growth or whole-body fish composition. Anyway, to the best of our knowledge, there seem to be no other studies considering the dietary inclusion of a non-axenic multi-species marine consortium in fish feeds. For these reasons, the comparison of the present results with previous research studies is not straightforward and should be done with caution. In any case, the results of the present study are consistent with the ones of Dallaire et al. (2007) as the dietary inclusion of microalgae biomass generally improved fish performance and feed intake, although only the fish fed with the 10% microalgae consortium (MC10) reached a significantly higher final body weight than the control diet. MC20 diet had a growth performance that did not differ from other treatments but resulted in a significant increase of FCR. Similarly, the rainbow trout fed with diets including 9.5% of a *Nannochloropsis* and *Isochrysis* blend or including also *Schizochytrium* exhibited significantly poorer FCR, a result not unlike those obtained by Walker and Berlinski (2011) on cod or Cardinaletti et al. (2018) on European sea bass.

For the above-mentioned reason, in the present study, a comparison with a test diet including monospecific dried biomass of *Nannochloropsis* spp. has been considered in the experimental design. *Nannochloropsis* sp. is a unicellular microalga with a polysaccharide cell wall (Hibberd 1981) and a promising ingredient in aquafeeds both as a successful fish oil substitute (Eryalçın and Yıldız 2015; Gbadamosi and Lupatsch 2018; Lozano-Muñoz et al. 2020) and the form of the defatted meal as an alternative to fish meal (Sørensen et al. 2017). Moreover, *N. oceanica* became better digested by European sea bass than other microalgae marine species (Batista et al. 2020b). In the present study, the replacement of terrestrial plant source by *Nannochloropsis* sp. dried biomass did not significantly affect diet palatability, fish growth performance, or biometric indices compared with C diet after a 75-day feeding period, confirming previous observation in the European sea bass fed with 8% of *N. oceanica* (Batista et al. 2020a). A recent study carried out by Valente et al. (2019) showed that the dietary inclusion up to 15% of defatted *Nannochloropsis* sp. biomass can replace fish meal in European sea bass diets without affecting fish growth performance and biometric indices. Moreover, Haas et al. (2016) showed that a 50 % fish oil replacement by *Nannochloropsis* sp. biomass did not hamper the growth performance of juvenile European sea bass. Other studies considering different dietary inclusion of *N. oceanica* indicate that moderate inclusion levels (<15 g/kg diet) do not affect growth and feed performance in other fish species like spotted wolffish (*Anarhichas minorhas*) and Atlantic salmon (*Salmo salar*) (Sørensen et al. 2017; Knutsen et al. 2019). On the contrary, higher dietary inclusion levels of *Nannochloropsis* sp. biomass hampered growth and feed conversion in Nile tilapia (Abdelghany et al. 2020) and Atlantic salmon (Sørensen et al. 2017; Teuling et al. 2018).

The nutritional value of a feed depends not only on its nutrient content but also on the animal’s ability to digest and absorb it. Consequently, the evaluation of the nutrient digestibility is the first step to determine the feasibility of using a microalgae product in aquafeeds (Allan et al. 2000; Tibbetts et al. 2006; Guedes and Malcata 2012; Cardinaletti et al. 2018). The effects of the dietary utilization of a microalgae consortium on zootechnical fish performance observed in the present study are similar to those of other studies where the dietary inclusion of microalgae biomass resulted in a marked reduction in nutrient and energy digestibility independently of the microalgae species studied, i.e., *T. suecica* (Tulli et al. 2012; Vizcaíno et al. 2016b), *Isochrysis galbana* (Tibaldi et al. 2015)*, Phaeodactlylum tricornutum* (Sørensen et al. 2016), and a blend of *Tetraselmis suecica* and *Tisochrysis lutea* (Cardinaletti et al. 2018). In the present study, the replacement of terrestrial plant source by 10% *Nannochloropsis* spp. did not significantly affect the nutrient and energy ADCs in European sea bass. The ADC values mirrored the data recently reported for defatted *Nannochloropsis* sp. in European sea bass (Valente et al. 2019), where dry matter, protein, lipid, and energy ADC figures were 79.9%, 94.2%, 97.1%, and 88% respectively when included at 10% of the diet in fish meal replacement. Similar data were observed by De Cruz et al. (2018) when a moderate (6%) dietary inclusion of *N. salina* did not significantly modify the protein ADC in comparison with a fish meal and fish oil-based diet in *Morone* sp., with a figure close to the one observed in the present experiment for MC10 (88.4% vs 90.0%). On the contrary, in post-smolts (215 g) Atlantic salmon, a 10% dietary inclusion of defatted *Nannochloropsis* sp. resulted in a significant decrease of dry matter (71.6% vs 76%), protein (85% vs 87.9%), lipid (88.6% vs 92.6%), and energy (81.5% vs 85.9%) ADCs as compared to a fish meal-based diet (Sørensen et al. 2017). Higher dietary inclusion levels (30%) also resulted in a significant decrease of nutrient and energy digestibility in comparison with the values reported for Atlantic salmon (67.3% for dry matter, 82.2% for protein, and 77.4% for energy ADCs, respectively) (Gong et al. 2018).

One of the parameters affecting dietary digestibility is the processing technology adopted to obtain the microalgae-based ingredient (Batista et al. 2020b). The microalgae cell wall is hard to digest and can limit the bioavailability of intracellular nutrients. A recent study carried out by Teuling et al. (2018) shows that cell wall disruption increases the bioavailability of *N. gaditana* biomass in the diet for Nile tilapia juvenile. The substantial inclusion of disrupted microalgae biomass (30%) resulted in an increase in protein and lipid ADCs from 62 to 78% and from 50 to 82%, respectively. Similar results were also observed by Tulli et al. (2017) with *Chlorella sorokiniana* dry biomass included in the diet for rainbow trout (*Oncorhynchus mykiss*).

Low nutrient digestibility has also been associated with intestinal morphological alterations such as reduction in the intestinal absorption area (Silva et al. 2015; Araújo et al. 2016; Moutinho et al. 2018), but the results of the present study do not support such conclusion as villus length and width remained similar among dietary treatments. Nevertheless, the inclusion of the microalgae consortium resulted in a significant decrease in the number of total acid goblet cells at the end of the feeding trial, suggesting lower intestinal protection against bacterial translocation (Torrecillas et al. 2019)*.* Increased neutral GC was recently associated with higher protein and energy ADC values in European sea bass fed with *N. oceanica* (Batista et al. 2020a), but this could not be observed in the present study. In addition, the intestinal structure and enzyme activities are determinant in nutrient absorption and provide a physical barrier against pathogenic microorganisms. Thus, this aspect merits further consideration even though the dietary treatments did not hamper gut functionality, herein considered a general framework of animal physiological welfare. Maltase and sucrase-isomaltase are disaccharidases belonging to hydrolases, which split the disaccharides into glucose and glucose and fructose, respectively. The activity of both disaccharidases in the terminal phase of the digestion of the carbohydrates was higher in pyloric caeca than in proximal and distal intestine. According to previous studies (Krogdahl et al. 1999; Harpaz et al. 2005; Tibaldi et al. 2006; Messina et al. 2019), the data observed in the present study confirm that pyloric caeca are the main site of the final digestion of the carbohydrates. In this part of the gut, only the test diet containing *Nannochloropsis* modulated the maltase activity. On the contrary, the distal part of the intestine seems to be affected by the presence of the algal consortium that stimulates the activity of both disaccharidases. Considering the decreasing amount of starch in the diets MC10 and MC20, it can be assumed that the small amount of starch provided by the consortium becomes available only in this tract of the intestine. γ-GT plays an essential role in the final digestion and absorption of proteins and is one of the main enzymes located in intestinal microvilli. γ-GT reached the highest activity in the PCs as also reported by Harpaz et al. (2005), who studied the response of Asian seabass *Latex calcarifer*, featuring a size similar to our experimental fish, to different feeding levels. On the contrary, Messina et al. (2019) and Tibaldi et al. (2006) for European sea bass and Harpaz and Uni (1999) for several fish species found that the distal intestine revealed the highest activity of γ-GT. It has to be highlighted that the size of the fish considered by these authors is much larger than that of the European sea bass considered in the present trial and that this specific feature could be related to differences in the enzymatic activity. The effect of MC10 and MC20 diets on the activity of γ-GT in the PC tract could be a consequence of the dietary protein quality, which also affected the protein digestibility. Alkaline phosphatase is an enzyme of the mature epithelial gut cells and is considered a marker of intestinal integrity. In the present study, the dietary inclusion of the consortium or *Nannochloropsis* biomass did not significantly affect the activity of ALP, indicating that microalgae did not cause major functional changes in gut integrity of European sea bass juveniles, as previously reported by Batista et al. (2020b) and Messina et al. (2019) for adult European sea bass and Vizcaíno et al. (2018) for sole. On the contrary, a previous study by Vizcaíno et al. (2014) showed that the inclusion of *Scenedesmus almeriensis* in the diet of gilthead seabream juveniles resulted in a quadratic response of ALP activity to increasing supplementation of algal biomass.

## Conclusion

This is so far the first study aimed at evaluating the dietary utilization of multispecific marine microalgae consortium biomass originated from a HRAP for a commercially relevant species. The results support a possible substitution of up to 10% of terrestrial vegetable ingredients by the microalgae consortium dried biomass with a significant increase of European sea bass final body weight, though impairing nutrient and energy digestibility. *Nannochloropsis* sp. biomass also has the potential to partially substitute terrestrial plant ingredient up to 10% of the diet without affecting growth performance, dietary nutrient utilization, and gut enzymatic activities. Algal consortium and *Nannochloropsis* sp. biomass could undergo specific processing techniques before being included in fish feed formulation to improve nutrient bioavailability. To increase aquaculture sustainability, this study using fish farm effluents to produce a multispecific marine non-axenic valuable biomass represents the first attempt to enhance a circular use of natural biomasses aquafeeds. Such an approach still needs further efforts, and the safety issues connected with their utilization need specific evaluations.
